# Poor-Law Infirmaries, Medical Education, and the People

**Published:** 1886-10-23

**Authors:** 


### POOR-LAW INFIRMARIES, MEDICAL EDUCATION, AND THE PEOPLE.

**Worthy of Imitation.** THE St. Pancras Guardians have this week resolved to make a most important departure in the administration of the Poor-law Infirmary under their charge. In response to a representation made by Mr. William Adams, F.R.C.S., the Chairman of the Infirmary Committee, and greatly to the credit of that gentleman, as well as of themselves, they have decided to appoint two assistant medical officers in the place of Dr. Bridgeford, resigned. The two assistants so appointed will hold office on conditions similar to those on which the house-physicians and house-surgeons hold their appointments at the general hospitals. It is to be presumed that the Local Government Board will not withhold its sanction from this proceeding, and if that presumption be correct, we may expect other Boards of Guardians to follow the example of the St. Pancras authorities with all convenient speed. In taking this step the Guardians of St. Pancras have shown an enlightened appreciation of important facts and problems. A step has been taken which may be fraught with the most beneficial results, not only to the patients at the Poor-law Infirmaries, but also to the medical profession, and still more to the public at large. There are whole worlds of facts connected with pauper hospitals, with general hospitals, with medical education, and with the medical profession generally, and the relations that all these bear to the people, which lie as completely beyond the ken of outsiders as do the ice-bound regions of the remotest north.

**More Knowledge Wanted.** The public as yet knows nothing and cares nothing about all these things. Nevertheless, they are subjects concerning which ratepayers and all other persons ought not only to have some knowledge, but to be well informed. To confine our remarks to Poor-law Infirmaries and to medical education, What are the facts? Speaking broadly, the patients at these infirmaries, who are numbered by tens of thousands, receive, under the existing system, the smallest possible modicum of skilled medical attention, and they suffer very greatly in consequence. On the other hand, newly qualified medical practitioners and the whole class of medical students, though possessed of valuable knowledge and time, have been prevented, up to the present, from bringing both to the rescue of the sick inmates of the Poor-law Infirmaries. This exclusion and seclusion has caused patients, practitioners, and students to suffer greatly in consequence. But who is it that really suffers most because of this divorce between neglected patients and eagerly acquisitive doctors? It is not the pauper patients, nor yet the disappointed doctors; it is the general public. By depriving the budding practitioner of these legitimate opportunities of practising his art, we keep down the general level of the medical skill which is hereafter to be exercised upon non-paupers, *i.e.*, upon ourselves.

**The Present System.** As a sample of what takes place under the present system, take the case of the St. Pancras Infirmary itself. In round numbers there is an average of 500 patients always in the wards. These may be divided almost equally into three classes, viz., acute, sub-acute, and chronic. Of these, the acute cases require to be seen every day, and some of them twice a day; the sub-acute every other day, and the chronic once a week. Given two medical officers for all these patients, as under the old system, it is seen that each officer must visit, at least, 130 persons daily, or eleven persons in each hour, and he must be at actual work for thirteen hours each day. Now that is a thing impossible to be done if each person's case is to be intelligently dealt with; and if each person's case is not to be intelligently dealt with, but to be slurred over, then the medical officer is encouraged to do his work dishonestly, and the patient is treated with positive cruelty, for he is given a mere pretence of a doctor instead of actual medical skill.

**Unite the Helpers to the Helpless.** Is there, then, no help for this state of things? There is; and the helpers are most eager to help, if not for the patients' sakes, at least for their own. The Poor-law Infirmaries are admirable fields for the best kinds of clinical work. The best class of newly qualified medical men are most eager to enter upon such fields, but hitherto the gates have been most obstinately shut against them. The

St. Pancras Guardians have now opened their gates; and it may be hoped that this is but a preliminary to a similar course of action on the part of Poor-law Guardians generally. In a word, the Poor-law Infirmaries should be affiliated to the large general hospitals having medical schools. There will thus be secured for each patient skilled and efficient medical attention; for a large number of meritorious men an eagerly sought and invaluable opportunity of attaining a higher degree of skill in their noble art; and, for the public generally, a larger class of medical practitioners of experience and practised ability as well as of sound scientific attainments. Mr. Ritchie, the President of the Local Government Board, is one of the most capable administrators of the day. In Mr. Ritchie's hands, therefore, with the able assistance of Dr. Buchanan, the St. Pancras seed is likely to grow rapidly into a sapling of fine proportions and much needed strength.

